# The Use of Silicone Simulators for Bile Duct Anastomosis Education in Medical Conferences for the Purpose of Improving the Canadian Medical Education Directives for Specialists (CanMEDS) Competencies

**DOI:** 10.7759/cureus.68131

**Published:** 2024-08-29

**Authors:** Rebecca Mosaad, Erica Patocskai, Adam Dubrowski

**Affiliations:** 1 Health Sciences, Ontario Tech University, Oshawa, CAN; 2 Surgical Oncology, University of Montreal, Montreal, CAN

**Keywords:** medical education, simulation in medical training, suturing techniques, knot tying techniques, bile duct anastomosis

## Abstract

This technical report explores the potential of including silicone bile duct simulators for the purpose of completing a bile duct anastomosis (BDA) in medical conferences. The purpose is to target the need for exposure to more surgical skills and to contribute to the Canadian Medical Education Directives for Specialists (CanMEDS) requirements, as per the Royal College of Physicians and Surgeons of Canada.

Data collection was completed at the 2023 Canadian Conference for the Advancement of Surgical Education (C-CASE) in Montreal, Canada. For several years, the quality improvement feedback received at the end of these conferences suggested a few areas of improvement, one of which was related to the concept of return on investment (ROI). The participants spend a considerable amount of funds to travel to the conferences but feel that the only measurable gains are at a research capacity and thus only relate to two CanMEDS competencies. By leveraging C-CASE, the aim is to enhance students' educational experience during events they already intend to attend. Initially, students participated in a five-part simulation workshop and engaged in a think-aloud protocol (TAO). From there, nine participants were recruited for a focus group to further understand the perceived educational value and feedback on both the simulators and the conference structure.

## Introduction

Simulation-based education (SBE), specifically in medical and healthcare training, allows the trainee to practice practical clinical skills before applying them to real patients [1]. Typically, this instruction is done in a laboratory setting with the appropriate simulated environments, simulation tools, and instructor guidance on the procedure or skill. This allows for the facilitation of teaching and learning of crucial competencies and skills necessary for healthcare professionals [2]. By providing the opportunity for students to participate in SBE, it encourages clinical error to be done in an environment where the patient’s safety and health are not at risk. It also encourages the development of hand-eye coordination, problem-solving, and decision-making skills, and enhances the ability to actively listen and collaborate with peers [3]. Further, the need to develop skills such as suturing and knot-tying techniques can be applied to a plethora of medical responsibilities and should be continuously honed.

The Royal College of Physicians and Surgeons of Canada has outlined a set of roles to define the responsibilities of a physician, more commonly referred to as the Canadian Medical Education Directives for Specialists (CanMEDS). Medical experts in Canada are expected to uphold clinical practices that are “up-to-date, ethical, and resource-efficient” [4]. CanMEDS outlines six roles (communicator, collaborator, leader, health advocate, scholar, and professional) to fulfill five core competencies. This paper focuses on competencies 3.3 and 3.4 under medical expert, which state: “Prioritize a procedure or therapy, taking into account clinical urgency and available resources,” and “Perform a procedure in a skillful and safe manner, adapting to unanticipated findings or changing clinical circumstance”, respectively.

Medical students, residents, and practicing medical doctors often participate in medical conferences as supplementary educational opportunities to their formal medical or practical training. Historically, these medical conferences are heavily research-focused and provide a minimal opportunity for hands-on learning [5]. Taking advantage of these events, workshops, and conferences that are already taking place provides students with an environment where they can enhance practical skills, such as suturing and knot-tying techniques, under professional supervision. Through this paper, we aim to understand if there is a practical opportunity to incorporate SBE in conferences, and how it improves the skills of the participants. This also targets a secondary gap, as it is well established that the Canadian healthcare system is unsustainable and has limited resources [6]. This solution would mean that instructors are not required to take additional time away from their practice to help train and educate students.

With this identified gap, we sought to understand if there is a potential solution that lies within these conferences for minimal exposure to practical surgical skills development. By integrating bile duct simulators, how do participants report progress to perceived self-efficacy outcomes, and can it be used to contribute to the improvement of the CanMEDS requirements?

## Technical report

Context

This annual conference was designed, organized, and run by members of the Canadian Undergraduate Surgical Education Committee (CUSEC), a committee contributing to the surgical education of undergraduate medical students. CUSEC members formed the Canadian Conference for the Advancement of Surgical Education (C-CASE) as an opportunity for medical students of all levels interested in surgical education to gain experience.

In a collaborative effort with the CUSEC Chair, Canadian Research Chair in Healthcare Simulation, simulation graduate students, and a digital designer, the simulators were provided as an in-kind contribution to the organizing committee. 

Several simulators were provided for the conference and set up with five different working stations, with approximately 20 placements per station. Each simulator, including the bile duct simulators, was isolated in their own rooms where participants could work collaboratively with those working on the same skill [7,8]. Assessments on acceptability and feasibility were only conducted on the bile duct simulators. Prior to beginning the hands-on technical sessions for learners, all participants were asked to complete a consent form and were provided time to raise any questions or concerns about the study. 

Medical students and residents were then provided approximately two hours to circulate the different simulation centers in 20-minute increments. During this time, a hepatobiliary surgeon circulated the bile duct anastomosis (BDA) station and supported students while they practiced.

Input

Building on previous work completed by colleagues [7,8], the simulator was initially engineered for the purpose of conducting a BDA. It was designed in collaboration with surgical residents from the University of Montreal (Montreal, Canada), and simulation graduate students from maxSIMhealth, situated at Ontario Tech University (Oshawa, Canada).

Through that project, it was concluded that the initial simulator required a few modifications based on conversations with surgeons. This included a thinner diameter for the wall, modifications to the length and thickness, a change in color, and finally, to make the simulators softer and more pliable [7,8]. These concerns were addressed in the newer iteration of the simulator - length was added to assist with ease of manipulation, a thinner wall diameter, a change in color to beige, and changing the type of silicone used to provide a more pliable result. The newer iteration of the simulator was utilized in the 2023 C-CASE BDA workshop.

Process and procedure

Qualitative Data Collection

Data for this technical report were collected using multiple qualitative methods. First, a think-aloud protocol (TAO) was employed with all conference participants. This protocol allows participants to verbalize their inner thoughts and provide real-time feedback on their experiences, especially during the use of the simulator. This approach helps capture higher-level thinking and minimizes recall bias, as participants communicate their thoughts with minimal prompting [7,8]. The TAO was facilitated by the researchers and a research assistant, who also took field notes during the sessions.

Additionally, a 25-minute focus group was conducted involving students in a semi-structured interview guided by predetermined questions. The focus group was recorded and later transcribed by the primary researcher. Field notes were also taken during the focus group to capture additional context and observations.

A simulation session quality improvement survey was distributed to all conference participants after the session. This survey followed the stop, start, change, and continue model, a recognized method in higher education for qualitative data collection [9]. Feedback relevant to the BDA and conference structure was included in the analysis.

Quantitative Data Collection

Quantitative data was collected through a follow-up survey provided to the focus group participants based on a modified version of the Michigan Standard Simulation Experience Scale (MiSSES) [10]. The survey comprised three main sections: self-efficacy, realism, and educational value, along with a section for freeform comments. Participants responded to nine questions using a five-point Likert scale, ranging from zero to five. The survey was administered via Google Forms (Google LLC, Mountain View, USA).

The survey data, considered ordinal, were presented as mean values. Standard deviation (SD) tests were performed due to the small number of participants and the objective of the analysis, which was to inform design improvements rather than validate findings [11].

Overall, the combination of qualitative and quantitative methods provided a comprehensive understanding of participant experiences and feedback, informing the ongoing development and refinement of the simulation sessions and conference structure.

Participants

Forty-four (n=44) medical students participated in the workshops and, by extension, the think-aloud portion of data collection. There were nine (n=9) participants in the focus group consisting of junior and senior medical students, which were snowball sampled from those participating in the workshops (n=44). Each of the focus group participants filled out a MiSSES survey. 

Outcomes

Qualitative Data Analysis

**Figure 1:**
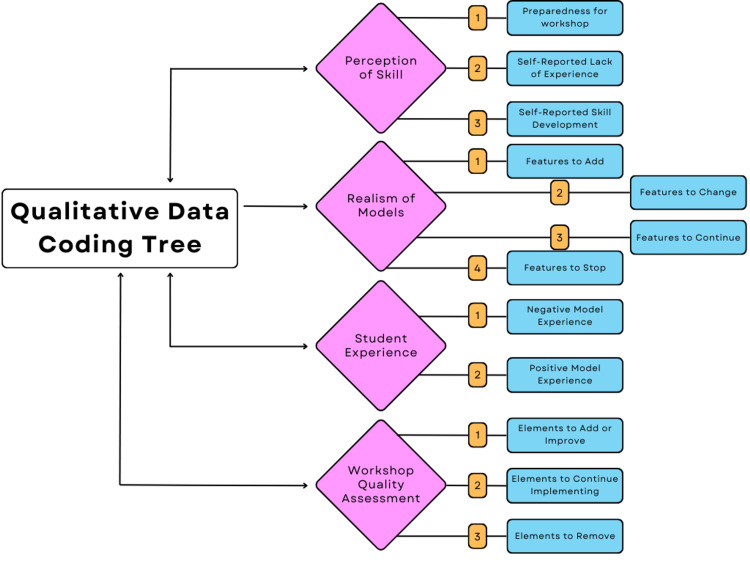
Qualitative data coding tree

All qualitative data was inductively coded by the primary lead author (RM). Following this, a one-hour consultation session with an external researcher to discuss codes brought to a consensus four main themes with corresponding sub-themes, as illustrated in Figure 1.

The qualitative data yielded comprehensive results on what both the simulators and the conferences should look like in the future. A codebook with definitions and theme inclusion criteria was developed inductively once the coding process began (Table 1). 

**Table 1:** Codebook utilized for all qualitative data

Major Theme	Sub-Theme
Realism of Simulators: Accuracy and realism of bile duct simulators.	Features to Add: Suggestions from participants to add to the simulators to improve quality, efficacy of training, or better user experience. Features to Change: Suggestions from participants to change the simulators to improve quality, efficacy of training, or better user experience. Features to Continue: Suggestions from participants to maintain features of the simulators and areas that did not require alterations or improvements. Features to Stop: Features that were not well perceived or did not resemble an accurate representation of the bile duct would need to be stopped in future developments.
Student Experience: General feedback on participant’s personal experiences during the workshops.	Positive Experience: Feedback reporting productive experience while utilizing the simulator. Negative Experience: Negative feedback in regards to the user's experience with the simulator.
Workshop Quality Assessment: Feedback that was provided was not specific to the bile duct simulator but rather to the workshop environment, coordination, or overall conference experience.	Elements to Add/Improve: Elements that were perceived positively by participants but required improvements in execution or additions to make the experience more all-encompassing and efficacious. Elements to Continue: Elements that were well perceived and did not require changes. Furthermore, aspects under this code are encouraged to be maintained in future reiterations of this conference or similar events.
Perception of Skills: Self-reported skill level before, during, and after the workshops.	Preparedness for Workshop: Student’s self-reported preparedness for the bile duct anastomosis workshop. Self-Reported Lack of Experience: Student’s self-reported feeling of being unprepared for the workshop. This includes but is not limited to inadequate previous experience with suturing or the procedure as a whole. Self-Reported Skills Development: Student’s self-report improvement in skills after participating in the workshop and practicing on simulators.

The realism of simulators: This section, also scoring low in the quantitative data set, suggested that modifications to the simulators would be essential to mimic an authentic bile duct. "It’s not slippery, because there’s liquid in the body, right, there’s fluid, so this is, the grip is good, but I don’t know if it's as much as a good grip in the human body" (Participant 1).

Furthermore, a number of participants described issues with poor stabilization of the simulators, a lack of proper surgical posture with the model’s setup, and the color of the thread being too difficult to see against the color of the simulator. Tools and environmental aspects did not mimic the surgical field precisely - for instance, students were seated and gloves were not provided at this conference. "[You] should always practice suturing with gloves because it changes the texture" (Participant 4). This suggests that more attention needs to be paid to the interaction between medical tools and the simulator. Again, consistently with the quantitative data, there was plenty of feedback on the continuation of allowing the use of simulators, despite the feedback on logistical modifications.

Student experience: In many instances during the TAO and the focus group, the observers noted, “When asking the students if they were having difficulties or restrictions from using the simulators, students would criticize their skills and lack of expertise as a reason for having difficulties with using the simulator” (field notes). Students and instructors alike did “appreciate that the simulators were tearable so that we can practice applying adequate force” (field notes). Students thoroughly enjoyed using the simulators describing the experience as "fun," "exciting," and "great for practice." One student even noted that it is a “good opportunity to learn the basics” (Participant 2).

Conference feedback: Recurring feedback on the conference structure revolved around the idea of (i) providing more time to practice with the simulators, and (ii) pre-instruction and guidance on how to complete the task. Students requested future sessions to be a “minimum of forty-five minutes” (Participant 1). A tutorial-style approach was suggested because: “You’ve [got] big jumps between what you can practice on your own and what you need facilitated” (Participant 2). Another student stated: “I also feel like the presence of a tutor, or many tutors, is the most beneficial. Because you can always go on your own and practice. Like what we are doing on our own, but when you have a teacher he can tell you, or she can tell you every single detail, these are too far apart, or closer together, or not symmetrical, or more pressure, less pressure, these things that you don’t know when you start off” (Participant 4). Again, 100% of feedback on whether the hands-on component of the conference should remain was reported as a feature that must stay.

**Figure 2:**
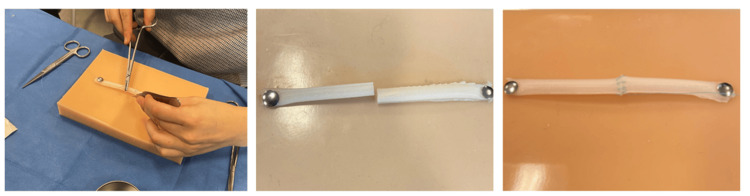
A student practicing BDA on the bile duct simulator (left). A bile duct simulator prior to the completion of BDA (middle). A bile duct simulator post-BDA (right). BDA: Bile duct anastomosis

Workshop preparedness and skills development: One of the comments suggested that some students were unprepared to practice this task: “We can’t compare with real bile ducts… because we haven’t done any suturing on bile ducts” (Participant 1). Two students followed up by stating they did not know how to begin repairing the simulator (Figure 2) and asked if coordinators “considered giving more specific training” (Participant 5). Unanswered questions about whether this procedure could be done laparoscopically and what a bile duct looks like suggested that students had no exposure to the procedure and were completely novel at it. Despite this, quite a few students reported that they felt more comfortable performing the procedure, and even with the minimal time they were provided to practice, their skills improved.

Quantitative Data Analysis 

**Figure 3:**
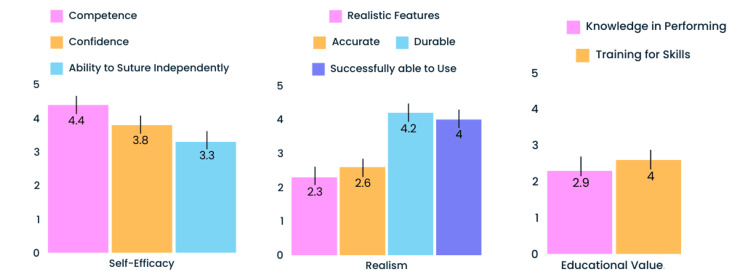
MiSSES survey mean results MiSSES: Michigan Standard Simulation Experience Scale

The quantitative results (Figure 3) from the MiSSES feedback survey indicated that the simulator had the potential to develop competence in performing a BDA (4.4 out of 5), confidence in performing a BDA (3.8 out of 5), and found that the simulator was a good training tool for performing a BDA (4.0 out of 5).

The realism of the simulator scored the lowest with realistic features (2.3 out of 5) and accuracy (2.8 out of 5) being the lowest. Notably, it was found that the bile ducts performed well in durability (4.2 out of 5) and ability to successfully use the simulator (4.0 out of 5).

Finally, participants reported knowledge in performing the skill (2.9 out of 5) and the ability to practice independently (3.3 out of 5). This was likely due to the fact that some of the sutures students were asked to practice in the session would typically be performed with a surgical assistant, which was not available in the simulation practice.

## Discussion

In this technical report, it was explored if the integration of SBE via bile duct simulators could be used to improve opportunities for students in order to fulfill their CanMEDS requirements. Similar work was conducted in the past with rural and remote (R&R) physicians in Canada [12], where the integration of fundamental skills training workshops contributed to their Mainpro+® Credits requirement [13]. Mainpro+® was designed by The College of Family Physicians of Canada to support the continuation of professional development [14]. This is crucial to the maintenance of certification and practice. This paper’s work differs as it targets a new demographic, student physicians (not limited to R&R), and instead contributes to the CanMEDS competencies, a requirement of all Canadian physicians. This new contribution of integrating SBE in conference settings is a new angle in creating opportunities for students to begin practicing and to prepare them for their future medical practice.

One of the findings was the importance of having formal instruction to explain how the procedure is to be conducted, proper techniques, as well as other medical knowledge required prior to completing a BDA. Thus, there should be a dedicated learning management system (LMS) including a curriculum for instruction and a method of evaluation of participants. It is important to maintain this “assessment” component as it may be a true indicator of obtained education [15]. This is doubly important as a large component of SBE is ensuring that the correction of mistakes is done prior to practicing on real patients. Without a guided experience, participants tend to learn the skill incorrectly and may not be able to transfer a skill to real practice [16]. Besides the limited surgeon supervision, this was something that was missing in this conference. Furthermore, since there was not a formal assessment of learning, any improvements that were documented were self-reported, which can be biased due to social desirability, and less reliable than a grounded assessment [17,18]. 

In the 2023 iteration of the C-CASE conference, the participants entered the workstations and were immediately provided time to practice, without instructions on how to proceed. However, in future iterations, students may be asked to complete a pre-conference asynchronous training to prepare them for the simulation experience. This would not replace supervisory support or correction during the workshops.

Another point of improvement is that participants were exposed to many workstations with limited time at each. Keeping in mind the limiting time factor of the conference itself and limited professional supervision, we propose longer workshop times, an increase in qualified supervisory support, or a remote learning assessment. This can be in the form of photographs or video recordings of the students' works, which would be submitted and assessed post-conference and later sent back to students with feedback. However, this would not replace immediate feedback, and supervisors are still encouraged to support and promote real-time learning [16]. This should be spearheaded in future iterations of the conference and explored in further investigation and research in this area of experiential learning.

Looking outwardly, although this work is being completed with Canadian institutions and students, this lack of education seems to be a widespread issue. A systematic review reported that only 24.7% of medical schools in the United Kingdom incorporate basic suturing skills in their medical school curriculum [19]. This proposes a potential need for international implementation, and with the findings of this study, a solution to the widespread educational gap at hand. We propose beginning with the integration of low-cost simulators, such as the silicone bile duct, into conferences, workshops, and similar events held for medical students and professionals.

Finally, as per the CanMEDs Guide, medical students preparing for the transition into residency require experience on how to "perform a simple procedure under direct supervision" [20] and have additional opportunities to gain exposure and refine their skills. While this pilot intervention is novel and focused on two areas of CanMEDS competencies, the further implications of this proposed program may target other areas of the CanMEDS roles. For instance, 5.1 states: “Recognize and respond to harm from health care delivery, including patient safety incidents” [20]. For instance, integrating contingency planning or education on how to handle emergency situations could be incorporated into the simulation education - sometimes referred to as EBAT (event-based approach to training) [16]. Again, this would require further investigation and professional input on what would accurately reflect a surgical environment.

## Conclusions

Given the results of this technical report, it is concluded that there is great potential for adding hands-on practice components to medical conferences such as C-CASE. This is a two-fold solution. Firstly, by acquiring simulators from research and development labs, as opposed to industry, it alleviates some of the costs associated with organizing a conference by providing an option for low-cost simulators. In collaboration with research and development labs, conferences can integrate the SBE and provide students with the necessary resources required for continuous practice and maintenance of surgical skills. This supports medical training and has great potential in surgical education outside of the formal curriculum. Future direction may focus on investigating feasible options for the manufacturing and distribution of simulators.

Secondly, this contributes to fulfilling the CanMEDS requirements, specifically, since there are high satisfaction levels in the simulator, improving student competence (i.e., skills, knowledge, and attitudes), ability to use the simulator, and an excellent training tool for learning how to perform a BDA. As previously mentioned, medical students preparing for the transition into residency will now have additional opportunities to gain exposure and refine their skills. The improvements mainly rely on the research and development labs to innovate simulators that emulate anatomy in a more realistic manner. In future iterations of the conference, it is strongly urged to maintain these opportunities for students, with the modifications outlined above. We also aim to advocate this alternate approach to structuring medical conferences. Since this work suggests a positive relationship with the improvement of the CanMEDS requirements for students attending conferences, it is recommended that conferences catering to medical students and residents consider this blended approach of research dissemination and hands-on practice. This is expected to be an excellent avenue to supplement formal education and support students working towards becoming medical experts.
